# The quantification of root exudation by an *in-situ* method based on root morphology over three incubation periods

**DOI:** 10.3389/fpls.2024.1423703

**Published:** 2024-08-16

**Authors:** Chengfu Zhang, Yinmei Cai, Qingxia Zhao, Tengbing He, Tianxu Mao, Tao Zhang, Limin Zhang, Weici Su

**Affiliations:** ^1^ Guizhou Institute of Mountain Resources, Guizhou Academy of Sciences, Guiyang, Guizhou, China; ^2^ College of Agriculture, Guizhou University, Guiyang, Guizhou, China; ^3^ Institute of New Rural Development, Guizhou University, Guiyang, Guizhou, China; ^4^ College of Forestry, Guizhou University, Guiyang, Guizhou, China; ^5^ Guizhou Academy of Testing and Analysis, Guizhou Academy of Sciences, Guiyang, Guizhou, China

**Keywords:** root exudates, fine roots, root diameter, root morphological traits, rhizodeposition

## Abstract

Investigating the quantity and spatiotemporal dynamics of metabolite release from plant roots is essential if we are to understand the ecological significance of root exudates in the rhizosphere; however, this is difficult to quantify. In the present study, we quantified *in situ* root exudation rates during three incubation periods (0–24, 24–48, and 48–72 h) and fine roots within four diameter ranges (<0.8, 0.8–1.0, 1.0–1.2, and 1.2–2.0 mm), and also measured nine morphological traits in the fine roots of *Pinus massoniana*. Higher root carbon (C) exudation rates were detected during the 0–24 h period. During the 0–24 h and 24–48 h periods, nitrogen (N) uptake rates were higher than N exudation rates, while during the 48–72 h period, N exudation rates exceeded uptake rates. As C exudation increased during 0–48h incubation period, the uptake of N tended to level out. We concluded that the 24–48 h incubation period was the most suitable for capturing root exudates from P. massoniana. The exudation of C from the roots was positively associated with root mass, length, surface area, volume, the number of root tips, and the root tissue density, when incubated for 0–24 h and 24–48 h. Furthermore, length-specific C exudation rates, along with N exudation and uptake rates, all increased as the diameter of the fine roots increased. The release of root exudates could be efficiently predicted by the fine root morphological traits, although the accuracy of prediction depended on the incubation period. Higher values for fine root morphological traits were generally indicative of higher nutrient requirements and tissue investment, as well as higher C exudation rates.

## Introduction

Root exudates, defined as plant-derived primary and secondary metabolites (Badri and Vivanco, 2009), can transfer passively from roots to the soil due to the large concentration gradient between the cell cytoplasm and soil solution, or can be actively exudated (Jones et al., 2004). Root exudates play an important role in driving soil processes, such as carbon (C) and nutrient turnover, by stimulating microbial activity (Meier et al., 2017; Chari and Taylor, 2022). Root exudates are estimated to represent 5–21% of all photosynthetically-fixed C in plants and trees (Haichar et al., 2014). Developing methods to determine specific amounts of root exudate may provide critical insights into the overall role of root exudates in biogeochemical processes occurring at the root-microbe-soil-interface.

Quantifying root exudation is very difficult due to the fact that exudates can be continually absorbed by microbes (Oburger and Jones, 2018; Proctor and He, 2017). In a previous study, Phillips et al. (2008) developed a method for collecting and measuring exudates in forest soils *in situ* based on a cuvette system. In this method, washed fine roots (< 2 mm diameter) were placed into glass cuvettes that were filled with sterile glass beads and a nutrient solution. Following a period of incubation, the trap solution from each cuvette was flushed, filtered and analyzed for non-particulate organic C. The duration of sampling and the time of day when the exudation sampling occurs can exert significant impacts on exudation rates when adopting the excavation/hydroponic exudation trap approach (Oburger and Jones, 2018). Phillips et al. (2008) reported that root exudation was best described by a logarithmic function over the first 24 h and was influenced by the duration of incubation due to the reuptake of exudates by roots. In 2009, the same group performed a solution culture experiment with loblolly pine (*Pinus taeda*) seedlings between 0 and 24 h, and concluded that net C exudation rates could be calculated as the change between the beginning and ending of a 9 h incubation period (Phillips et al., 2009); analysis revealed that the reuptake time for the exudates by roots was 9 h. In another study, Williams et al. (2021) assessed the quantity and quality of root exudates in different ‘root recovery’ periods and found that a recovery period after root washing of at least 3 days was critical when collecting exudates because it prevented experimental bias caused by root damage. These findings indicate that the precise mechanisms underlying the variation in root exudation in relation to incubation period have yet to be elucidated. Thus, it is necessary to conduct further *in situ* collection experiments of root exudates from different species to determine the specific equilibrium and collection periods required.

Many studies have demonstrated a clear link between nitrogen (N) availability and C exudate. For example, a previous study reported that roots released a greater amount of labile C into soils with a higher inorganic nitrogen content (Yin et al., 2014). A ^15^N isotope-labeling experiment revealed that root exudation rates were significantly and negatively correlated with soil inorganic N uptake rates, as well as with the uptake rates of NO_3_^−1^-N and glycine-N (Liese et al., 2018). The release of C-rich exudates from the roots of plants is known to drive microbial decomposition processes, stimulate the decomposition of recalcitrant C, and enhance N-cycling in the soil (Yin et al., 2013). Few studies have directly linked specific root C exudation rates with specific N uptake rates (Liese et al., 2018). An approach for capturing soluble root exudates *in situ* may help us to investigate the relationship between root C exudation rates and N uptake rates. In this study, the cuvette was filled to saturation with N-containing nutrient solution to create an *in situ* method for the acquisition of C exudation. In addition to C-rich components, roots also release N. In the presence of N-containing nutrient solution, root N exudation or uptake may represent a consequence of bidirectional N flux. This possibility warrants further investigations to specifically quantify the relationship between C exudation and N uptake or exudation.

Fine roots are known to exhibit high levels of physiological activity and a faster turnover than coarse roots (>2 mm diameter) and are often used to investigate root exudates (Akatsuki and Makita, 2020). Previous research has demonstrated that root morphology can influence exudation rates (Nguyen, 2003). For example, studies involving ectomycorrhizal woody species revealed that root exudation rates increased with increasing specific root length (SRL) and specific root surface area (SRA), but decreased with increasing mean diameter (Akatsuki and Makita, 2020). Exudation rates from the roots of grassland and crop species have been negatively correlated with SRL but positively correlated with root diameter (Wen et al., 2019; Williams et al., 2022). Different root sizes are also known to exhibit heterogeneous physiological and chemical properties (Akatsuki and Makita, 2020). Researchers have yet to identify how root sizes <2 mm in diameter can influence root exudation.

*P. massoniana*, a premium species of timber, has been extensively cultivated in China due to its significant role in both water conservation and C sequestration (Deng et al., 2020). In the present study, we investigated root exudation and the morphological traits of *P. massoniana* over three incubation periods. The specific aims of our study were as follows: (i) to quantify the variation in exudation rates over three incubation periods and determine the equilibrium and collection periods; (ii) to quantify the relationship between C exudation and N uptake or exudation, and (iii) to identify the role of morphological traits, including diameter, root mass, root length, root volume, Number of root tips (NRT), SRL, SRA, and root tissue density (RTD), in predicting and explaining the variation in root exudation rates.

## Materials and methods

### Site description

This study was performed in a *P. massoniana* experimental plantation located in Guiyang, Guizhou Province, China (106°398″E, 26°2815″N, altitude 1145 m). The study region has a subtropical monsoon climate with mean annual temperatures of 14.9°C and mean annual precipitation of 1178.3 mm. The *P. massoniana* plantation, at the commencement of the study, was approximately 35 years old. The tree density and canopy density of *P. massoniana* were 853 stem hm^-2^ and 0.8, respectively. Additionally, the average diameter and height of *P. massoniana* were 32.2 cm and 28.32 m, respectively. The soil at the site was classified as Oxisol by The United States Department of Agriculture (USDA), and developed from red clay during the Quaternary period. More detailed descriptions regarding the site characteristics, refer to Zhang et al. (2022).

### Root exudates and root morphology measurements

Exudates were systematically collected in January, July, October, and December 2020, as well as April 2021, utilizing a culture-based cuvette system for *P. massoniana* (Phillips et al., 2008). Three 10 × 10 m plots were established and two mature trees with similar growth conditions per plot were selected. Three terminal fine roots (< 2 mm diameter) attached to each target tree were extracted from the topsoil layer (0–10 cm) with extreme caution. The total number of replicates is eighteen (3 cuvettes × 2 trees × 3 plots). After triple washing with deionized water to eliminate soil particles, the cleaned roots were delicately placed into root cuvettes, each consisting of a 30 mL sterile syringe filled with 1-mm diameter sterile glass beads (acid-washed by 2 M HCL). To guarantee the thorough elimination of C from the cuvette prior to the experimental incubation period, each cuvette was saturated with a C-free nutrient solution (0.2 mM K_2_SO_4_, 0.3 mM CaCl_2_·2H_2_O, 0.1 mM KH_2_PO_4_, 0.2 mM MgSO_4_·7H_2_O,and 0.5 mM NH_4_NO_3_) and flushed using a vacuum pump. This process was repeated three consecutive times. After the third flush, the syringes were injected with a C-free nutrient solution to meet the nutrient requirements for normal root growth. The cuvettes were then covered with aluminum foil and reintroduced into the original plot soil environment. Additionally, the cuvettes with glass beads and nutrient solution, but without roots were included as controls. Subsequently, nutrient solutions containing exudates in the syringe were collected by flushing the same syringe three times with a C-free nutrient solution after 24 h, 48 h, and 72 h, employing an automatic electric vacuum pump, with an additional nutrient solution reinjected into the system each day. Root exudation rates, as reported by Phillips et al. (2008), were found to be minimally influenced by C accumulation in the cuvette during a 24 h incubation period, spanning the diurnal cycle. After collection, exudates were filtered through a filter (0.22 μm) and stored in a refrigerator (-20°C). Analysis of root C and N exudates was performed using a TOC-TN analyzer (Vario, Germany). In addition, roots in each syringe were harvested, scanned with an Epson scanner (Seiko Epson Corporation, Japan), and morphological variables (i.e., root length, root surface area, root volume, and root tips) were analyzed using a WinRHIZO Pro 2019a instrument (Regents Instruments Inc., Quebec, Canada). Subsequently, the roots were oven-dried at 65°C and weighed. SRL (cm g^-1^) and SRA (cm^2^ g^-1^) were calculated as dividing root length and root surface area by root dry mass. RTD (g cm^-3^) was calculated as dividing root dry mass by root volume.

**Figure 1:**
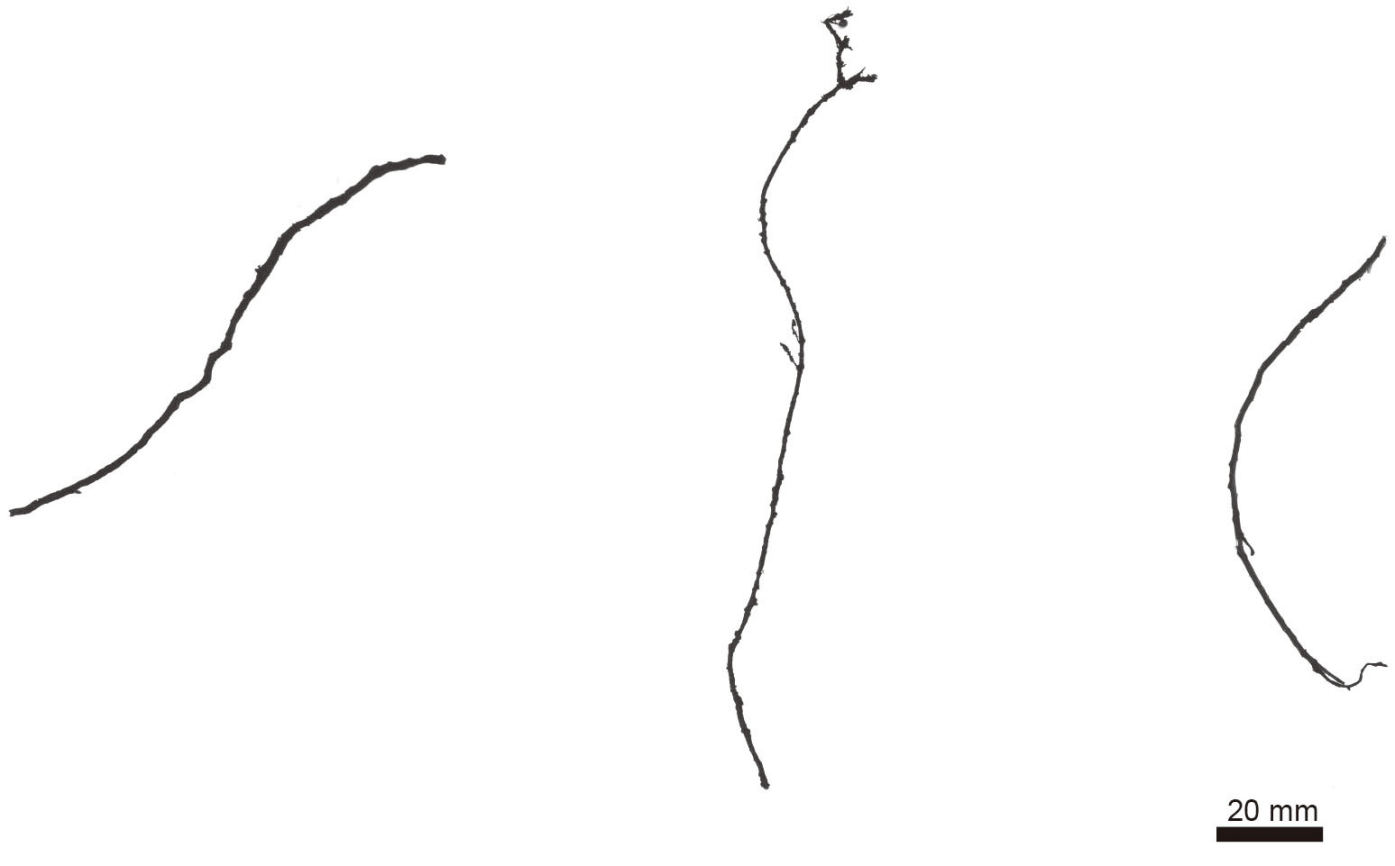
The morphological traits of fine root samples in the syringe.

We harvested 90 fine root samples from the syringe after collecting root exudates on five sampling dates. All intact root cluster samples were scanned at a resolution of 300 dpi using a calibrated flatbed scanner coupled with a lighting system for image acquisition (Seiko Epson Corporation, Japan). Images were analyzed using the WinRHIZO Pro 2019a instrument (Regents Instruments Inc., Quebec, Canada) to determine the average diameter of each root sample. The 90 fine root samples were classified into four diameter classes (<0.8 mm, 0.8–1.0mm, 1.0–1.2mm, 1.2–2 mm) based on the mean diameter of intact root clusters within the syringe (**Figure 1**). We obtained 27, 30, 21, and 12 fine root samples with mean diameters in the range of <0.8mm, 0.8–1.0mm, 1.0–1.2mm, and 1.2–2.0 mm, respectively. We calculated the rates of mass, length, and surface area-specific C or N exudation for each diameter class of fine root samples.

Total quantity of C and N exudation were calculated as the mass of C (μg) and N (μg) flushed from each root system (minus the average C and N quantity of the control cuvettes) over the 0-24 h, 24-48h and 48-72h incubation periods. Mass-specific C or N exudation rates (μg C or N g^-1^ root biomass h^-1^), length-specific C or N exudation rates (μg C or N cm^-1^ root length h^-1^), and surface area-specific C or N exudation rates (μg C or N cm^-2^ root area h^-1^) were calculated by dividing the total amount of C or N flushed from the root system by the total fine root mass, root length, and root surface area, respectively. Total quantity of C and N accumulation in a cuvette in 24, 48, and 72h incubation times were calculated as the mass of C (μg) and N (μg) flushed from each root system over the 0–24 h, 0-48h, and 0-72h incubation periods.

### Statistical analyses

Before analysis, the data underwent normality and variance homogeneity tests. A one-way ANOVA was also performed to examine the effects of incubation periods or diameter on root exudation rates. The study utilized the least—significant difference (LSD) test to evaluate significant differences. Statistical significance was established at a *P*-value below 0.05. Spearman correlation analysis was used to determine the correlation between root exudation and root traits. Principal components analysis (PCA) was used to show incubation periods effects on root exudation rates. All statistical analyses were performed with the Social Sciences (SPSS) software, version 22.0 (SPSS, Inc., Chicago, IL, USA).

## Results

### Variation in root exudation rates and the accumulation of exudates in response to different incubation periods

**Figure 2:**
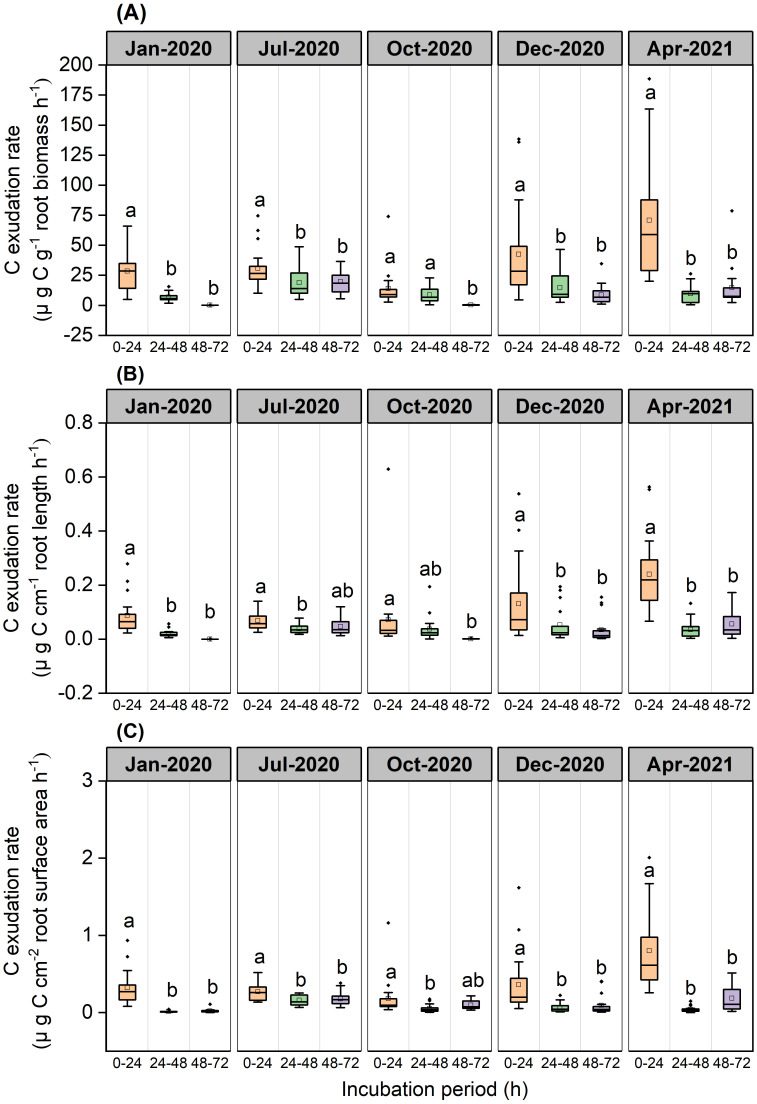
Boxplot of root C exudation rates for the *P*. *massoniana* across five sampling dates under different incubation periods (n=18). **(A)** Mass-specific C exudation rates. **(B)** Length-specific C exudation rates. **(C)** Surface area-specific C exudation rates. The central box in each boxplot indicates the median and the 25–75th percentile interquartile range. The whiskers extend to 1.5× the interquartile range or to the minimum and maximum value. Solid circles are outliers. Different lowercase letters indicate significant differences among the different incubation periods (*P <*0.05).

Exudation rates of C by roots varied significantly across incubation periods (*P*<0.001, **Figure 2**). Mass-specific root C exudation rates from the 0–24 h period were significantly higher than those from the 24–48 h and 48–72 h incubation periods in January (*P*<0.001), July (*P*=0.012 and *P*=0.019, respectively), December 2020 (*P*=0.001 and *P*<0.001, respectively), and April 2021(*P*<0.001) (**Figure 2A**). Mass-specific root C exudation rates from the 0–24 h and 24–48 h periods were significantly higher than those from the 48–72 h period in October 2020 (*P*<0.001 and *P*=0.015, respectively) (**Figure 2A**). Length-specific root C exudation rates from the 0–24h period were significantly higher than those from the 24–48 h and 48–72 h periods in January (*P*<0.001), December 2020 (*P*=0.018 and *P*=0.004, respectively), April 2021(*P*<0.001), and were significantly higher than those from the 24–48 h period in July 2020 (*P*=0.002), and significantly higher than those from the 48–72 h period in October 2020 (*P*=0.017) (**Figure 2B**). Surface area-specific C exudation rates from the 0–24h period were significantly higher than those from the 24–48 h and 48–72 h periods in January (*P*<0.001), July (*P*<0.001 and *P*=0.004, respectively), December 2020 (*P*<0.001 and *P*=0.002, respectively), and April 2021(*P*<0.001), and were significantly higher than those from the 24–48 h period in October 2020 (*P*=0.017) (**Figure 2C**).

**Figure 3:**
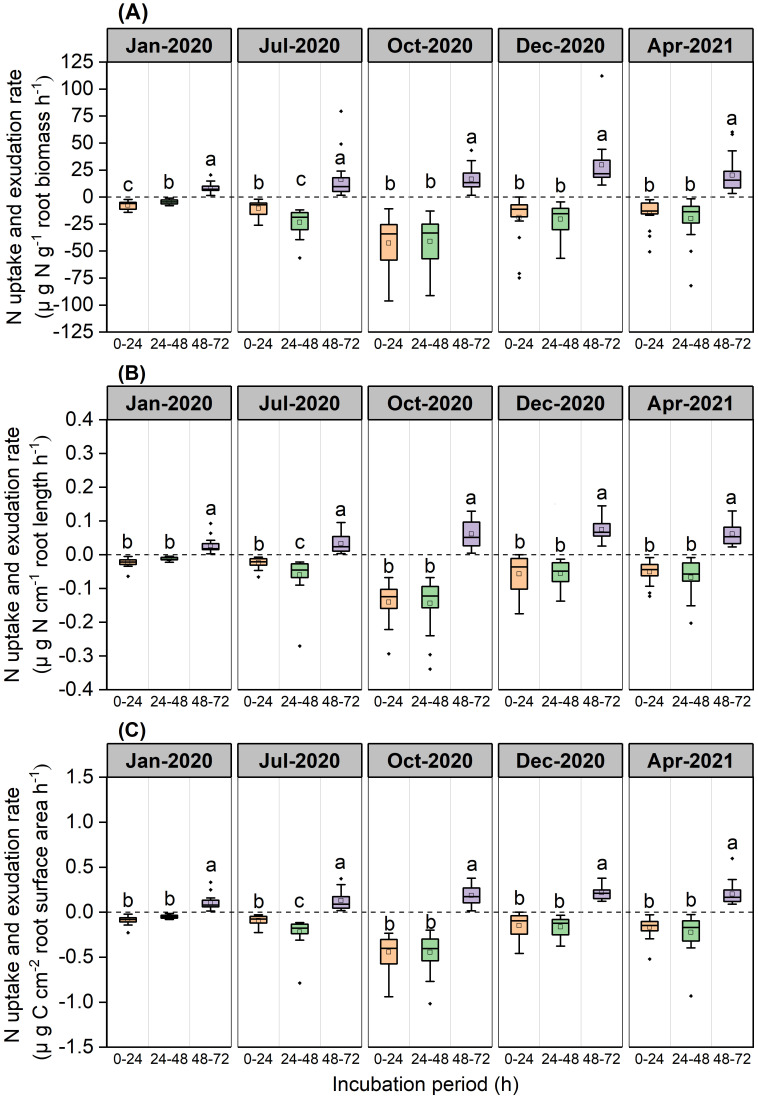
Boxplot of root N uptake and exudation rates for the *P*. *massoniana* across five sampling dates under different incubation periods (n=18). **(A)** Mass-specific N uptake and exudation rates. **(B)** Length-specific N uptake and exudation rates. **(C)** Surface area-specific N uptake and exudation rates. The central box in each boxplot indicates the median and the 25–75th percentile interquartile range. The value is negative, indicating that root N uptake rates. The value is positive, indicating that root N exudation rates. The whiskers extend to 1.5× the interquartile range or to the minimum and maximum value. Solid circles are outliers. Different lowercase letters indicate significant differences among the different incubation periods (*P <*0.05).

Over the 0–24 h and 24–48 h periods, root N exchange was negative, thus indicating that N uptake rates were higher than N exudation rates (**Figure 3**). During the 48–72 h period, root N exchange was positive, thus indicating that exudation rates were higher than uptake rates (**Figure 3**).

**Figure 4:**
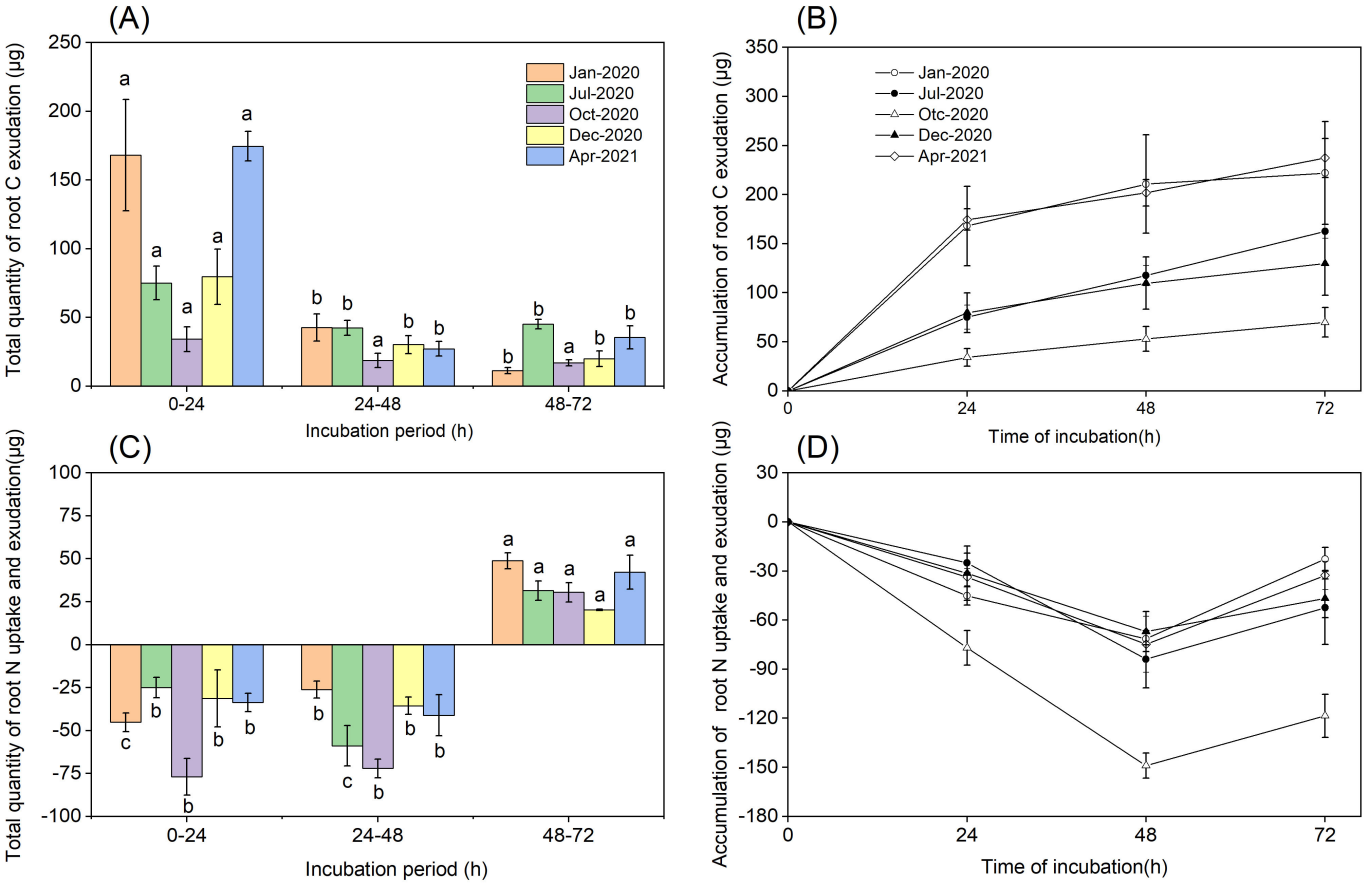
Total quantity and accumulation of root exudation. **(A)** Total quantity of root C exudation. **(B)** The accumulation of root C exudation. **(C)** Total quantity of root N uptake and exudation. **(D)** The accumulation of root N uptake and exudation. Error bars are ± SE of the mean (n=3). For **(A, C)**, Different lowercase letters indicate significant differences among the different incubation periods.

We evaluated the total quantity and accumulation of C and N in a cuvette to determine the equilibrium point at which C and N efflux was balanced by the reuptake of exudate by the roots of *P. massoniana*. Based on these measurements, we concluded that the total quantity of C released from roots over the 0–24 h period was higher than those over the 24–48 h and 48–72 h periods (*P*<0.001, **Figure 4A**). C accumulation rates increased linearly over a 0-24 h period during all seasons, but declined between 24 and 72 h (**Figure 4B**). The total quantity of root N uptake was higher than the total quantity of root N exudation over 48 h (**Figures 4C, D**). We concluded that N uptake rates increased up to 48 h in all seasons, but declined between 48 and 72 h. The equilibrium point at which N uptake from N-containing nutrient solution was balanced by the N efflux from *P. massoniana* roots was between 48 and 72 h.

**Figure 5:**
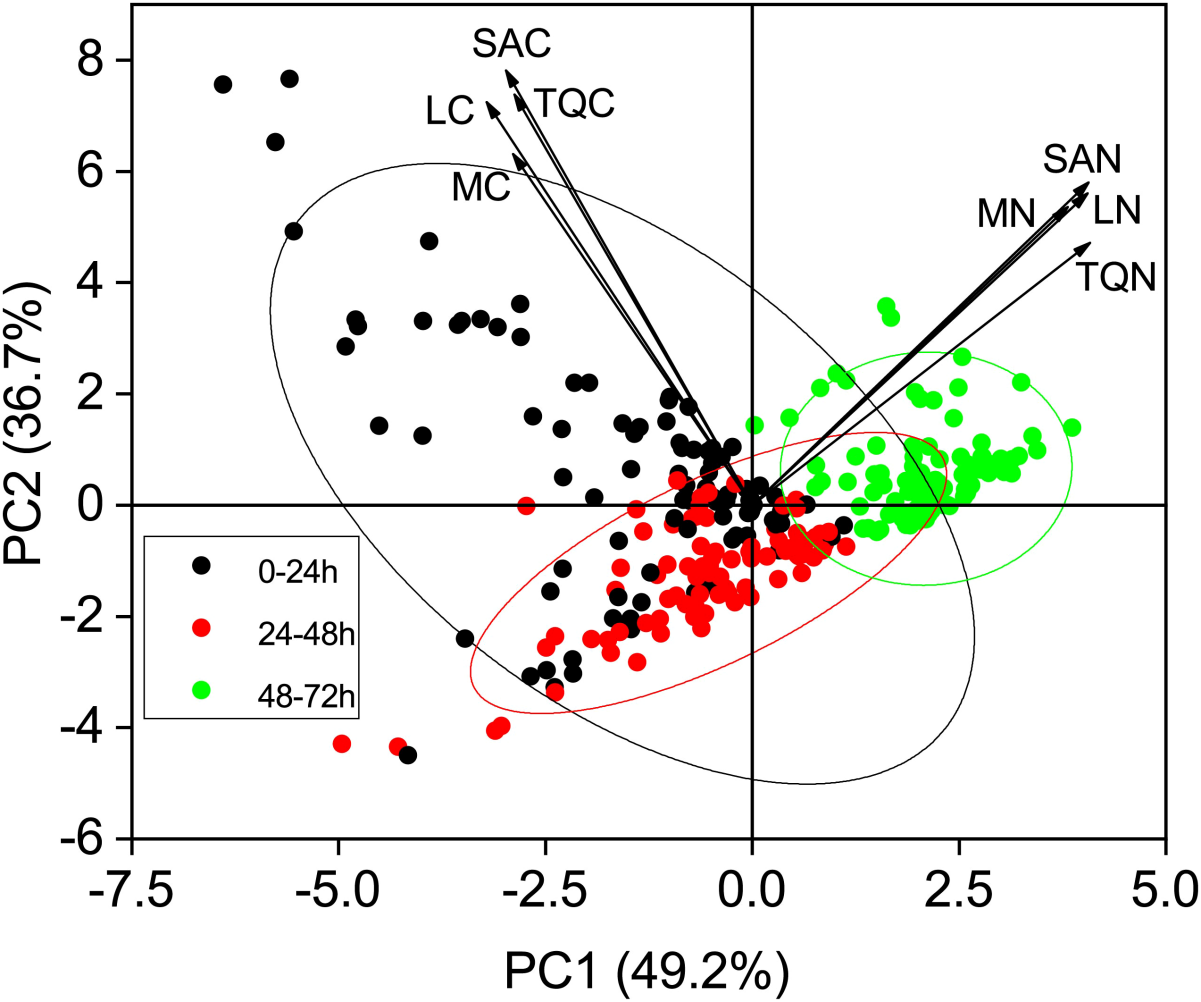
Principal component analysis (PCA) score plots of root exudate profiles over three incubation periods. Arrows on PCA indicate projections for each individual trait, and values in parentheses on the axes are total explained variance. Ellipses represent the 95% confidence interval of three incubation periods on the two dimensions. MC, mass-specific C exudation rates; LC, length-specific C exudation rates; SAC, surface area-specific C exudation rates; MN, mass-specific N exudation rates; LN, length-specific N exudation rates; SAN, surface area-specific N exudation rates; TQC, total quantity of C exudation; TQN, total quantity of N exudation.

Principal component analysis (PCA) based on exudation rates and quantity across five sampling seasons and three incubation periods explained 85.9% of the variation in the first two components (**Figure 5**). PCA of root exudation revealed clear separation by incubation periods. The first component (PC1) represented 49.2% of the variability, and the second component (PC2) represented 36.7% of the variance (**Figure 5**). Furthermore, axis 2 clearly shows separation for the 24-48 h period, particularly with regards to the exudation rate and quantity of C, thus suggesting that there was a reduction of C concentration during this period (**Figure 5**).

### Correlations between root C exudation and root N uptake and exudation

**Figure 6:**
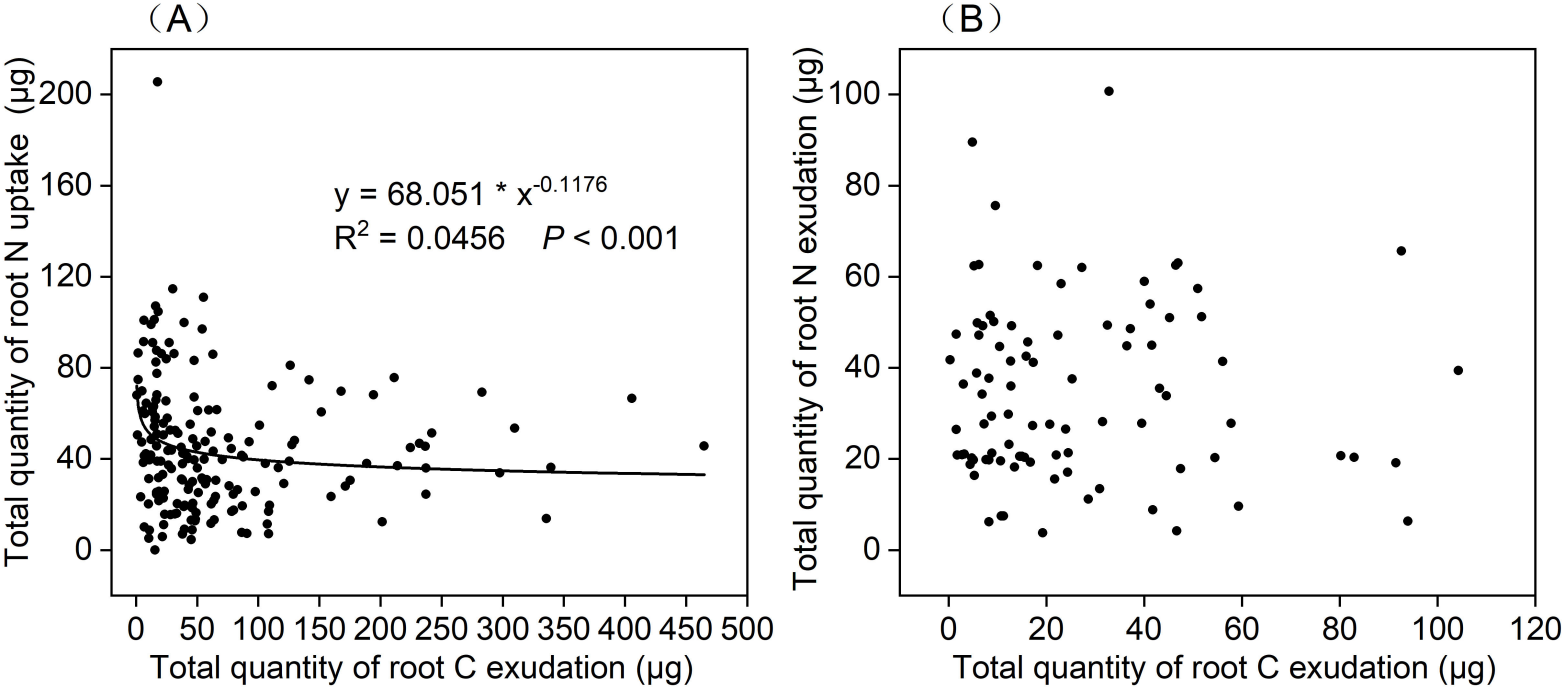
Relationship between the total quantity of root C exudation and **(A)** total quantity of root N uptake under 0–24 h and 24–48 h incubation periods(n=180), **(B)** total quantity of root N exudation under 48–72 h incubation period(n=90).

Next, we evaluated the relationship between the total quantity of root C exudation and the total quantity of root N exudation and uptake in the cuvette over periods of 0–24 h, 24–48 h, and 48–72 h (**Figures 2**–**4**). Analysis of the relationship between the total quantity of root C exudation and the total quantity of root N uptake from the 0–48 h incubation period was best described by a power function (y = 68.051 * x^-0.1176^; R^2^ = 0.0456; *p* < 0.001; **Figure 6A**). The total quantity of root C exudation between 48–72 h did not follow a specific relationship with the total quantity of root N exudation (**Figure 6B**).

### Correlations between root exudates and the morphological traits of roots

**Figure 7:**
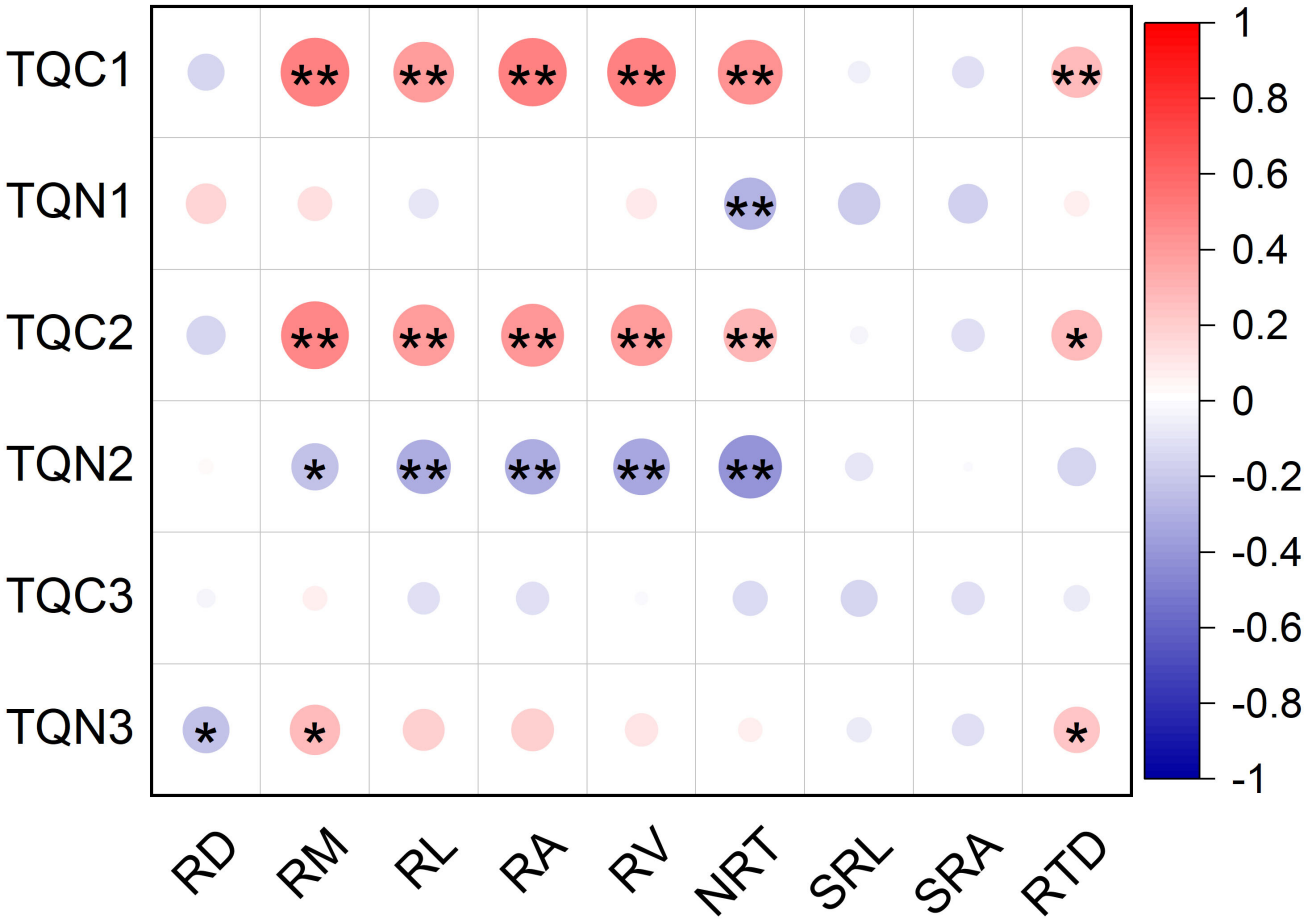
Relationship between the total quantity of root exudation and root morphological traits of <2mm diameter class of *P*. *massoniana*. (n=90). Asterisk represents a significant correlation, *p < 0.05, **p < 0.01. TQC1, total quantity of root C exudation in 0–24 h; TQN 1, total quantity of root N uptake in 0–24 h; TQC2, total quantity of root C exudation in 24–48 h; TQN2, total quantity of root N uptake in 24–48 h; TQC3, total quantity of root C exudation in 48–72 h; TQN3, total quantity of root N exudation in 48–72 h; RD, Root diameter(mm); RM, Root mass(g); RL, Root length(cm); RA, Root surface area(cm^2^); RV, Root volume (cm^3^); NRT, Number of root tips; SRL, Specific root length (cm g^-1^); SRA, Specific root surface area (cm^2^ g^-1^); RTD, root tissue density (g cm^-3^).

The total quantity of root C exudation from the 0–24 h and 24–48 h periods was positively related to root mass (*R^2^ = *0.49 and *R^2^ = *0.48, respectively; *P*<0.001), length (*R^2^ = *0.38 and *R^2^ = *0.39, respectively; *P*<0.001), surface area (*R^2^ = *0.48 and *R^2^ = *0.41, respectively; *P*<0.001), volume (*R^2^ = *0.49 and *R^2^ = *0.39, respectively; *P*<0.001), NRT (*R^2^ = *0.43 and *R^2^ = *0.3, respectively; *P*<0.001 and *P*=0.0042, respectively), and tissue density (*R^2^ = *0.27 and *R^2^ = *0.27, respectively; *P*=0.0089 and P=0.0010, respectively) (**Figure 7**). The total quantity of root C exudation from the 48–72 h period was not related to the morphological traits of roots (**Figure 7**).

The total quantity of root N uptake from the 0–24 h period was negatively correlated with NRT (*R^2^* =-0.28; *P*=0.0071; **Figure 7**). The total quantity of root N uptake from the 24–48 h period was negatively associated with root mass (*R^2^* =-0.23; *P*=0.027), length (*R^2^* =-0.31; *P*=0.003), surface area (*R^2^* =-0.32; *P*=0.0025), volume (*R^2^* =-0.33; *P*=0.0013), and NRT (*R^2^* =-0.41; *P*<0.001) (**Figure 7**). The total quantity of root N exudation from the 48–72 h period was positively correlated with root mass (*R^2^ = *0.26; *P*=0.012) and tissue density (*R^2^ = *0.23; *P*=0.033), but was negatively correlated with root diameter (*R^2^* =-0.23; *P*=0.031) (**Figure 7**). Root exudation was not correlated with either SRL or SRA (**Figure 7**).

### The effect of root diameter on root exudation rates under different incubation periods

**Figure 8:**
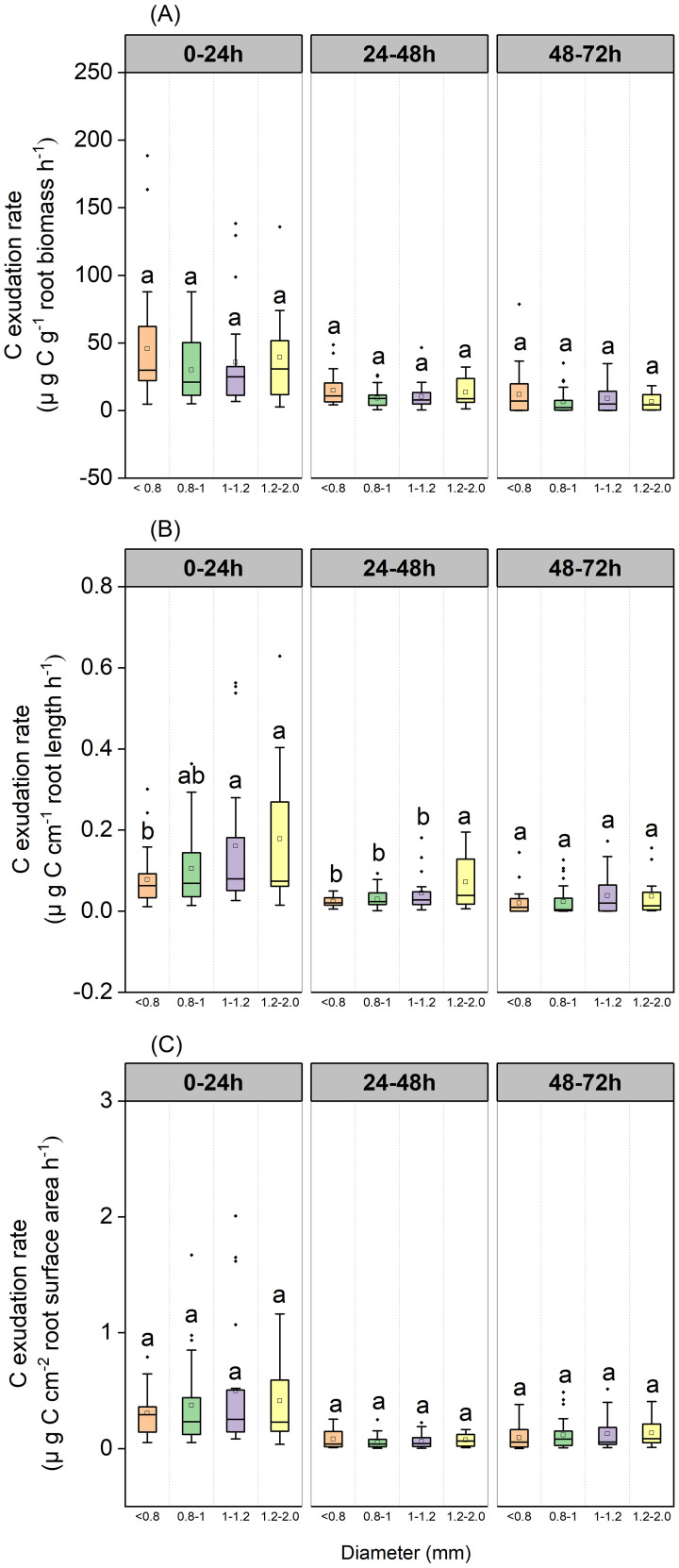
Boxplot of root C exudation rates over three incubation periods for the *P*. *massoniana* under different root diameter classes (<0.8 mm, n=27, 0.8–1.0mm, n=30, 1.0–1.2mm, n=21, 1.2–2 mm, n=12). **(A)** Mass-specific C exudation rates. **(B)** Length-specific C exudation rates. **(C)** Surface area-specific C exudation rates. The central box in each boxplot indicates the median and the 25–75th percentile interquartile range. The whiskers extend to 1.5× the interquartile range or to the minimum and maximum value. Solid circles are outliers. Different lowercase letters indicate significant differences among the different incubation periods (*P <*0.05).

Our results indicated that root diameter only had a significant effect on length-specific C exudation rates; however, this effect depended on the period of incubation (**Figure 8**). Length-specific C exudation rates from roots with a mean diameter in the range of 1.0–1.2mm and 1.2–2.0 mm were significantly higher than those from roots with a mean diameter <0.8mm during the 0–24 h incubation (*P*=0.029 and *P*=0.025, respectively, **Figure 8B**). Length-specific C exudation rates from roots with a mean diameter of 1.2–2.0 mm were significantly higher than those from roots with a mean root diameter < 0.8mm (*P*<0.001), 0.8–1.0mm(*P*<0.001), and 1.0–1.2mm(*P*=0.036) during the 24–48 h period (**Figure 8B**).

**Figure 9:**
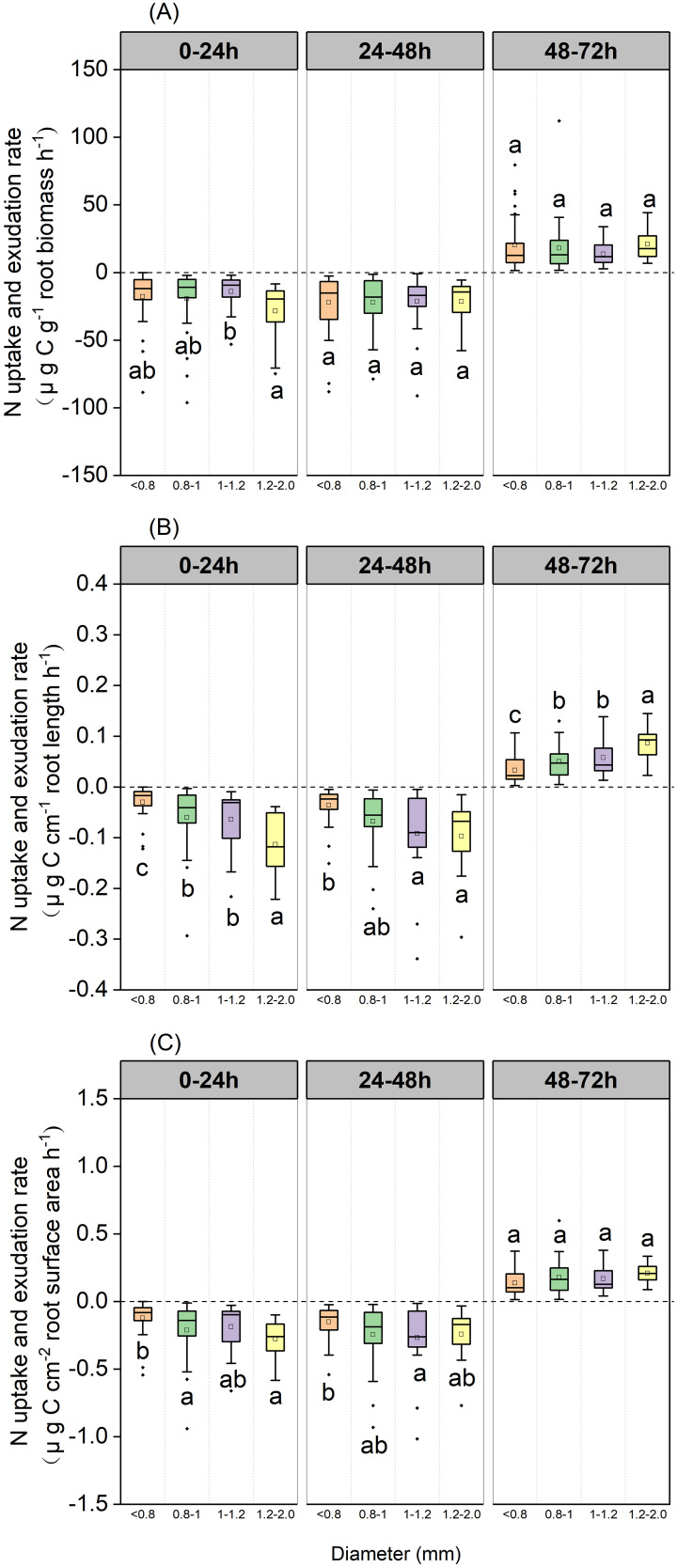
Boxplot of root N uptake and exudation rates over three incubation periods for the *P*. *massoniana* under different root diameter classes (<0.8 mm, n=27, 0.8–1.0mm, n=30, 1.0–1.2mm, n=21, 1.2–2 mm, n=12). **(A)** Mass-specific N uptake and exudation rates. **(B)** Length-specific N uptake and exudation rates. **(C)** Surface area-specific N uptake and exudation rates. The central box in each boxplot indicates the median and the 25–75th percentile interquartile range. The whiskers extend to 1.5× the interquartile range or to the minimum and maximum value. Solid circles are outliers. Different lowercase letters indicate significant differences among the different incubation periods (*P <*0.05).

Mass-specific N uptake rates did not differ when compared between various diameter classes when sampled after 48 h, although the 1.2–2 mm diameter class exhibited values that were significantly higher than the 1–1.2 mm diameter class when sampled after 24 h (*P*=0.048, **Figure 9A**). Length-specific N uptake rates for the 1.2–2.0 mm diameter class were significantly higher than for the <0.8, 0.8–1.0, and 1.0–1.2 mm classes when sampled after 24 h (*P*=0.029, *P*=0.024 and *P*<0.001, respectively, **Figure 9B**), and the uptake rates for the 1.0–1.2 and 1.2–2.0 mm diameter classes were significantly higher than those of the <0.8 mm diameter class when sampled after 48 h (*P*=0.003 and *P*=0.007, respectively; **Figure 9B**). Surface area-specific N uptake rates for the 0.8–1.0 and 1.2–2.0 mm diameter classes were significantly higher than those for the <0.8 mm diameter class when sampled after 24 h (*P*=0.048, *P*=0.011, respectively, **Figure 9C**), while those for the 1.0–1.2 mm diameter class were significantly higher than those of the <0.8 mm diameter class when sampled after 48 h (*P*=0.048 **Figure 9C**). Length-specific N exudation rates for the 1.2–2.0 mm diameter class were significantly higher than those for the <0.8, 0.8–1.0, and 1.0–1.2mm diameter classes when sampled after 72 h (*P*=0.047, *P*=0.010 and *P*<0.001, respectively, **Figure 9B**). Mass- and surface area-specific N exudation rates did not differ between diameter classes (**Figures 9A, C**).

**Figure 10:**
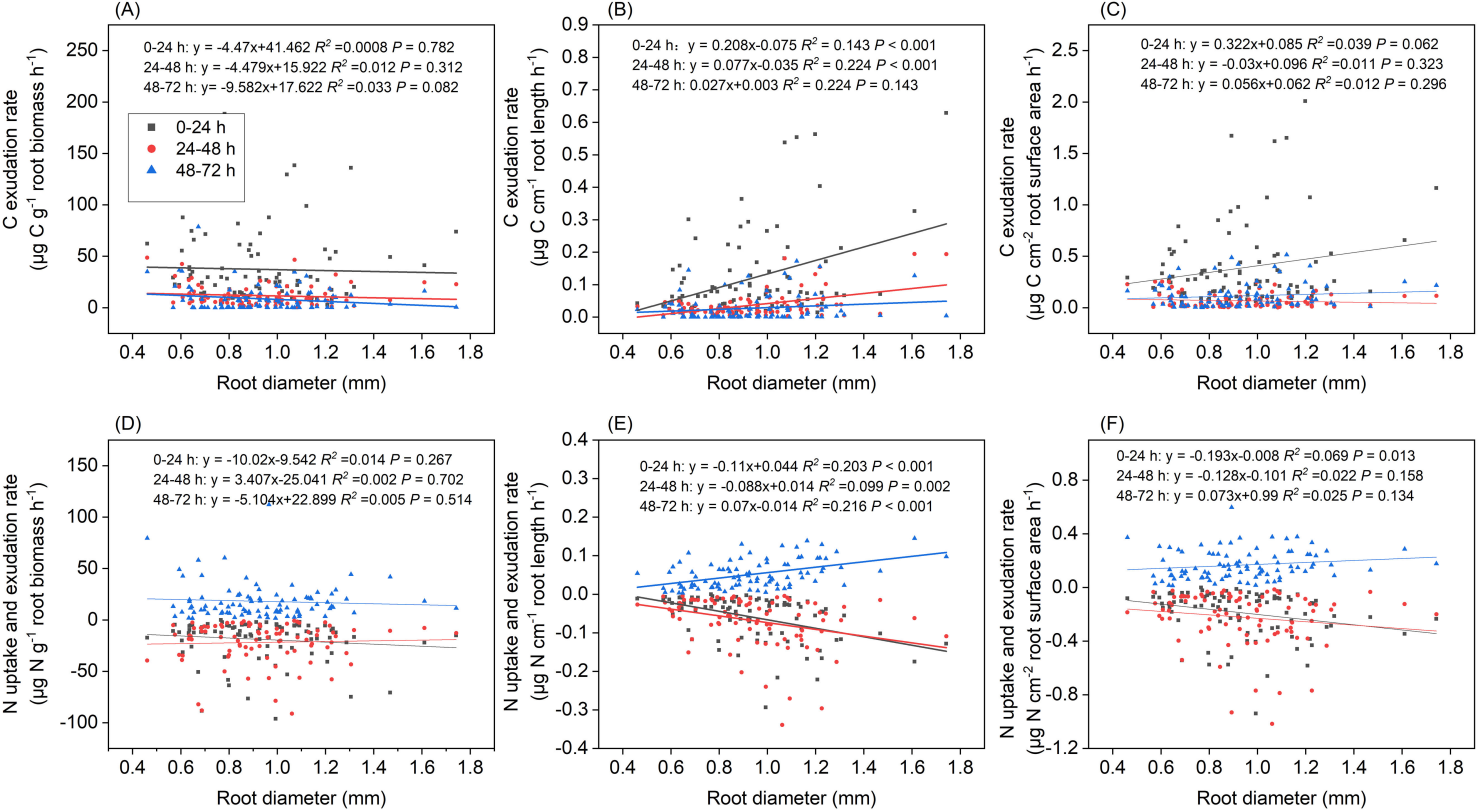
Relationship between root diameter and **(A)** mass-specific C exudation rates, **(B)** length-specific C exudation rates, **(C)** surface area-specific C exudation rates, **(D)** mass-specific N uptake and exudation rates, **(E)** length-specific N uptake and exudation rates, **(F)** surface area-specific N exudation rates under different incubation periods (n=90).

**Figure 11:**
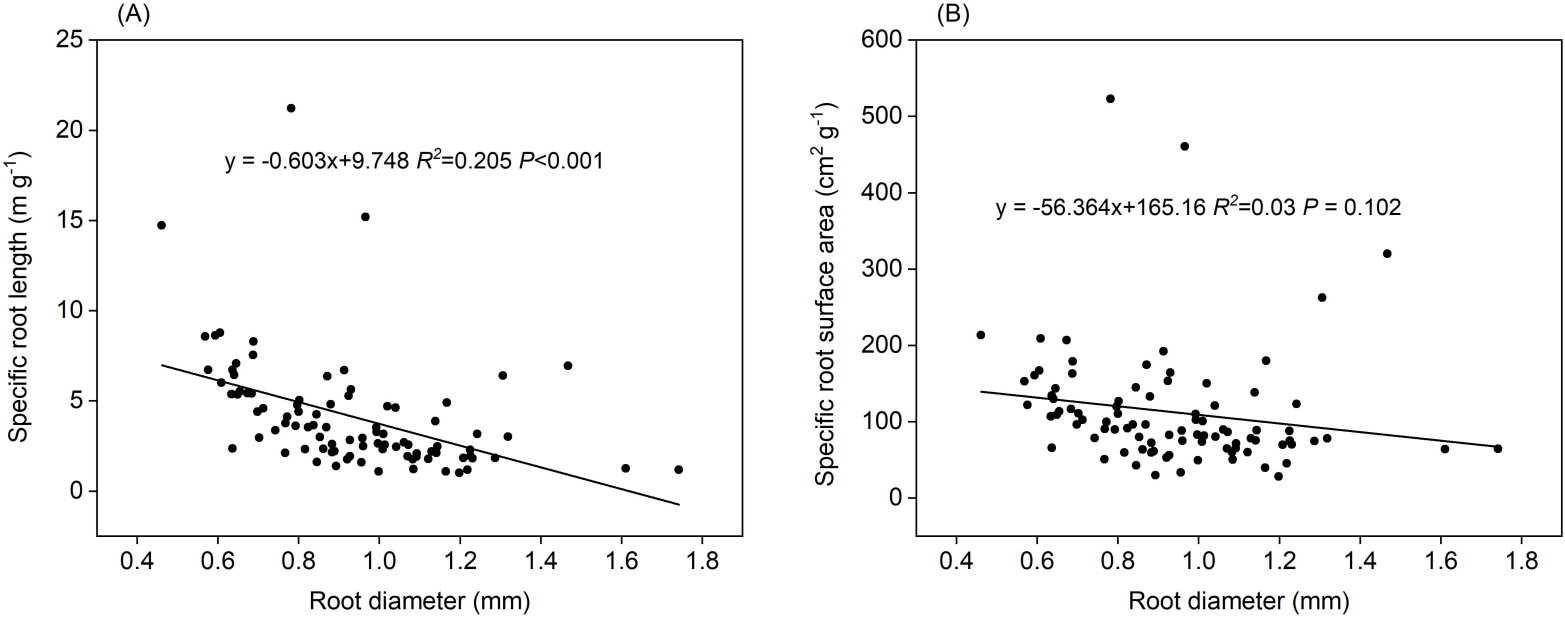
Relationship between root diameter and **(A)** specific root length, **(B)** specific root surface area (n=90).

Length-specific C exudation rates during the 0–24 h and 24–48 h periods, and length-specific N exudation rates during the 48–72 h period were significantly and positively correlated with root diameter (*P*<0.001, **Figures 10B, E**). Positive correlations of length-specific N uptake rates with root diameter were observed during the 0–24 h and 24–48 h periods (*P*< 0.001 and P=0.002, respectively, **Figure 10E**). The surface area-specific N uptake rates from the 0–24 h period were significantly and positively correlated with root diameter (P=0.013, **Figure 10F**). Mass- and surface area-specific C exudation rates, along with mass-specific N uptake and exudation rates, were not related to root diameter (**Figures 10A, C, D**). A significant and negative correlation was observed between root diameter and SRL (*P* < 0.01, **Figure 11A**) along with a weak correlation between root diameter and SRA (*P*=0.102, **Figure 11B**).

## Discussion

### The effects of incubation period on root exudation

Our analysis revealed that root C exudation rates during the 0–24 h period were significantly higher than those during the 24–48 h and 48–72 h periods (**Figure 2**). Root exudation rate is known to be determined by the concentration gradients of soluble organic compounds between the cytoplasm in the roots and the soil solution (Farrar et al., 2003; Jones et al., 2004). The exudation rates of C during the 0–24 h period were greater due to higher diffusion gradients between the root and the solution. Despite replacing the solution every 24 h to maintain the initial gradient, the release of higher molecular weight C compounds from the roots resulted in progressively lower diffusion gradients during the 24–48 h and 48–72 h periods. We interpret these declining diffusion gradients as evidence of reduced exudation rates during the 24–48 h and 48–72 h periods. Furthermore, it is likely that the roots reacquired a fraction of the exudate during the period of incubation. Roots recapture root exudates by utilizing various HC-ATPase-driven proton cotransporters (Jones et al., 2005). The reuptake of exudates has been reported for wheat (*Triticum turgidum*) and maize (*Zea mays*) (De Graaff et al., 2007), maize (*Zea mays*), rye grass (*Lolium multiflorum*), medic (*Medicago truncatula*) (Phillips et al., 2006), and loblolly pine (*Pinus taeda*) seedlings (Phillips et al., 2009). Furthermore, microorganisms in the soil can absorb and assimilate C from root exudates. Roots are known to be extensively colonized by mycorrhizal fungi and rhizoplane bacteria (Phillips et al., 2008); these microbial communities could have removed C efflux from roots during the 24–48 h and 48–72 h incubation periods. Thus, it is important to capture the C exuded prior to microbial assimilation. In addition, it is possible that some mycorrhizal fungal hyphae may become severed when the roots are separated from the soil; this would inevitably influence the flux of C from roots (Phillips et al., 2008). In such cases, cells that have sloughed away from the cells of broken fine roots and/or root hairs could result in initial C concentrations that are higher than after some days of equilibration (Oburger and Jones, 2018). Exudation profiles could inevitably be altered by the extension of incubation time and/or the physical damage to the roots caused by excavation and washing procedures (Oburger and Jones, 2018). A previous study reported that roots need some time to recover after excavation prior to the collection of exudates for research (Oburger and Jones, 2018). Considering our data, we suggest that the 24–48 h incubation period was most suitable for capturing soluble root exudates from the intact root systems of *P. massoniana* in the field based on a static culture-based system. We also consider that roots need a 1-day equilibration period to recover after excavation.

During the 0–24 h and 24–48 h periods, N uptake rates were higher than the N exudation rate, while during the 48–72 h period, root N exudation rates exceeded root N uptake rates (**Figure 3**). Roots acquire nutrients and therefore deplete N from N-containing nutrient solution to satisfy their N requirement, partly due to the separation of the roots from the soil (Farrar et al., 2003). Thus, the rate of root exudate influx exceeds the rate of efflux. In addition, the roots absorbed N from N-containing nutrient solution due to the large concentration gradient between the solution and the root cytoplasm during the 0–24 h and 24–48 h incubation periods. After the roots absorbed a sufficient amount of N from the N-containing nutrient solution, the concentration gradient in the root cytoplasm was greater than that of the culture solution. The unchanged N flux seasonal pattern also indicated that root N uptake and exudation were mainly controlled by the concentration gradient rather than the season.

### The correlation between C exudation and N uptake

In our study, correlation analyses showed that as C exudation increased, the uptake of N tended to level out (**Figure 6A**). In a previous study, Yin et al. (2014) detected greater levels of root exudation in ectomycorrhizal tree species than in arbuscular mycorrhizal tree species, along with greater organic to inorganic N content ratios in the soil. Greater exudation rates may indicate that trees have to pay a higher cost to extract nutrients from the organic matter in soil (Yin et al., 2014). Roots respond to low N uptake rates by increasing C exudation rates in order to optimize N acquisition (Liese et al., 2018). Conversely, during periods of high N uptake, roots reduce their C exudation to prevent the unproductive loss of C (Liese et al., 2018). In addition, low specific C exudation rates with simultaneously high specific N uptake rates may indicate that trees invest more C in releasing chelators that mobilize N from the soil (Liese et al., 2018). In our study, we measured only net instead of gross rates of C exudation and N uptake. Further examination of root exudation by C and N tracers across a broader range of N levels and further investigation of the observed relationship between root exudation and N uptake would be a worthwhile focus for future studies.

### Root morphology affect root exudation

In the present study, we found that root C exudation exhibited positive relationships with root mass, length, surface area, volume, NRT, and RTD during the 0–24 h and 24–48 h periods (**Figure 7**). Our findings are consistent with previous studies for woody species which reported that root exudation could be predicted efficiently by the consideration of root morphology (Phillips et al., 2008; Gao et al., 2024). Williams et al. (2022) previously explained that root exudation forms part of an exploitative resource uptake strategy and that the rate of root exudation would be positively correlated with exploitative growth-associated root traits. Individual plants with a larger root length and surface area may imply increase nutrient requirements or exhibit a higher surplus of C release, especially during seasons associated with rapid growth (Gao et al., 2024). RTD refers to the construction cost of the roots, and a higher RTD generally indicates higher levels of tissue investment (Weigelt et al., 2021). Root exudates are energy-rich substrates and carbohydrates that promote microbial activity to decompose organic matter in the soil (Meier et al., 2017). The resulting greater availability of nutrients, in turn, stimulates the construction and proliferation of root. Several studies have reported that root tips represent the active sites of exudation (Neumann et al, 2009) and are the preferential sites for root exudation (Li et al., 2018). Furthermore, mycorrhizal tips may represent both a source and sink for exudates (Gao et al (2024)). Notably, the correlation between organic acids in exudates and NRT is more pronounced than with other morphological root parameters (Li et al., 2018), thus emphasizing the preferential role of root tips in determining the rate of exudation.

There was no significant relationship between root exudation and either SRL or SRA. Our understanding of how root exudation covaries with SRL and SRA remains inconclusive. Previous studies have reported that root exudation showed positive (Meier et al., 2020; Wang et al., 2021), negative (Wen et al., 2019; Honvault et al., 2021) or no (Sell et al., 2022) relationships with SRL and SRA. Meier et al. (2020) reported that a doubling of SRL exponentially increased the exudation rates of mature trees; this is because root systems with a high SRL have a smaller diameter and thinner roots on average (Ma et al., 2018), a lower cortex:stele ratio (Kong et al., 2014), and reduced arbuscular or ectomycorrhizal fungal colonization (Kong et al., 2014; Brundrett and Tedersoo, 2018). Other researchers detected a significant and positive correlation between root exudation rate and SRA in ectomycorrhiza tree species (*Pinus densiflora* and *Larix. kaempferi*); this is due to the higher C load from fine roots with a higher SRA (Sun et al., 2021). HoweverGao et al (2024) reported that the relationship between root exudation rates of three tree species and SRL and SRA depends on seasonality. Thus, how root SRL and SRA interact with root exudation has still to be elucidated.

### Fine roots with a thicker diameter exhibited higher exudation rate

We found that length-specific C rates during the 0–24 h and 24–48 h incubations, length-specific N exudation rates during the 48–72 h incubation, and length-specific N uptake rates during the 0–24 h and 24–48 h incubations increased with higher root diameter (**Figures 8B**, **9B**, **10B**, **E**). These findings were consistent with previous studies, which showed that roots with a larger diameter may lead to higher rates of exudation in 18 common grassland species (Williams et al., 2022). Roots with a larger diameter incur higher construction costs for the roots because the roots exude substances or take up more N from N-containing nutrient solution to build roots. This could also explain why length-specific N uptake rates were higher for larger diameter roots. We didn’t see these same patterns for surface area-specific exudation rates. It is likely partially explained by the higher surface area of thicker roots for the same amount of root length. However, some previous studies found that smaller-diameter roots may be associated with higher rates of exudation (Akatsuki and Makita, 2020). A smaller root diameter provides less cortex habitat for mycorrhizal fungal symbionts, and has lower C costs for root construction and mycorrhizal symbiosis, a greater amount of C may be available for root exudation (Guo et al., 2008; Meier et al., 2020). Roots with a smaller diameter exhibit a high SRL and N content, and a shorter lifespan, contributing to the uptake of water and nutrient (Comas et al., 2002; Comas and Eissenstat, 2004; Luke McCormack et al., 2012). Roots with a smaller diameter probably relied predominantly on higher SRL or SRA to explore soil nutrient, whereas thicker roots were probably more reliant on higher root C exudation rates to stimulate soil organic matter decomposition and N or phosphorus mineralization (Wen et al., 2019). In the present study, we conducted measurements of *in situ* root exudation using an intact root segment (mean diameter: 0.94 mm) and thus deviated from the more conventional approach of employing small-scale intact root segments (Akatsuki and Makita, 2020). The intact root segment of larger diameter exhibited higher exudation rates due to its greater surface area compared to small-scale intact root segments of the same total root length. Consequently, a more nuanced understanding of root systems and their diverse diameter distributions is crucial for accurate predictions of exudation dynamics.

## Conclusions

Our analyses found that root exudation rates varied significantly across three incubation periods, and that the 24–48 h incubation period was considered as suitable for capturing soluble root exudates. In addition, we found that root C exudation and root N uptake during nitrate incubation periods were best described by a power function in order to optimize N acquisition and prevent the non-productive loss of C. Furthermore, our analyses showed that root C exudation rates were positively related to the morphological traits of roots and that fine roots with a thicker diameter had high exudation rates, although this depended on the period of incubation. We demonstrated that the release of root exudates was well-predicted by root mass, root length, root surface area, root volume, NRT, and RTD. Higher values for fine root morphological traits are generally indicative of higher nutrient requirements and tissue investment, suggesting that plants release higher concentrations of C from root exudates to build roots.

## Data Availability

The original contributions presented in the study are included in the article/supplementary material. Further inquiries can be directed to the corresponding authors.
